# Surveillance of a *PLOD* gene variant linked to fragile foal syndrome in Silesian horses in Poland: implications for genetic monitoring and breeding strategies

**DOI:** 10.2478/jvetres-2025-0060

**Published:** 2025-10-27

**Authors:** Monika Stefaniuk-Szmukier, Aleksandra Błaszczak, Bogusława Długosz, Adrianna Musiał, Katarzyna Ropka-Molik

**Affiliations:** Department of Animal Molecular Biology, Department of Farm Animal Biodiversity Conservation and Horse Breeding, National Research Institute of Animal Production, 32-083 Balice, Poland; Department of Farm Animal Biodiversity Conservation and Horse Breeding, National Research Institute of Animal Production, 32-083 Balice, Poland; Department of Animal Reproduction, Anatomy and Genomics, University of Agriculture in Kraków, 30-059 Kraków, Poland

**Keywords:** autosomal recessive disease, equine genetic disorders, genetic testing, PCR-RFLP, single nucleotide polymorphism

## Abstract

**Introduction:**

The Silesian horse is a heavy warmblood breed developed in Polish Silesia through the covering of local mares by East Frisian and Oldenburg stallions. Because of its historical significance and genetic heritage, the breed is part of a conservation programme in Poland. One of the genetic disorders of concern in warmblood horses is fragile foal syndrome (FFS), an autosomal recessive disease caused by a mutation in the *PLOD1* gene (c.2032G>A). Affected foals either perish in late pregnancy or are born with severe connective tissue abnormalities, leading to early death. As carriers do not exhibit symptoms, genetic testing is crucial for responsible breeding. This study aimed to assess the prevalence of the *PLOD1* mutation in the Silesian horse population.

**Material and Methods:**

Samples of DNA from 284 breeding horses were analysed using PCR and restriction-fragment length polymorphism and validated by Sanger sequencing.

**Results:**

The detected carrier frequency was 14.6%, an increase over previously reported carriage for this breed. Compared to other warmblood breeds, the carrier frequency in Silesian horses was higher than in Swedish Warmbloods, similar to the frequency in Hanoverians (14%) and also aligned with that in Oldenburg horses, from which Silesians historically derive.

**Conclusion:**

The results highlight the need for continued genetic monitoring and informed breeding strategies to prevent the spread of FFS in the Silesian horse population.

## Introduction

European equine diversity includes a variety of breeds developed in local regions and geographical areas which are currently under protection because of their cultural and historical value. These breeds, which exemplify national breeding traditions, have been developed over centuries, primarily by mating local mares with imported stallions to improve utility and meet the practical needs of different times.

The Silesian horse is a representative of the group of heavy warmblood horses. The current form of the breed was developed at the turn of the 19^th^ and 20^th^ centuries in the Silesian region through the covering of local mares by East Frisian and Oldenburg stallions. It is characterised by great draft power, good movement at the walk and trot and a lively temperament and gentle character. Silesian horses are currently bred for two purposes: some to compete at world-class level in driving and others to be used in recreational equestrian sports. Their excellence in high-level competition is proven by numerous medals won at the Driving World Championships (10, 14).

Genetic disorders in horses are of great importance from a breeder’s perspective, particularly if they have the potential to result in significant economic losses. One such disorder is fragile foal syndrome (FFS) caused by a single nucleotide polymorphism in the *PLOD1* (procollagen-lysine-2-oxoglutarate-5-dioxygenase1) gene (*PLOD1*:c.2032G>A, p.Gly678Arg) (15). It is considered an autosomal recessive disease, so only foals inheriting this variant from both parents will show the characteristic symptoms, which are death in the later stages of pregnancy or non-viability at birth with abnormal, hyperextensible fragile skin and hypermobility of joints (2, 6, 9). Carriers of the mutation do not show any symptoms, which makes genetic testing crucial for responsible breeding programmes to prevent the birth of affected foals. Several sources suggest the presence of FFS carriers in warmblood breeds from various countries, as well as in Thoroughbreds. Since Silesian horses trace their lineage back to Oldenburg warmbloods and have been improved with Thoroughbreed crossbreeding, they may have the mutation causing FFS. Silesians are included in the genetic resource conservation programme in Poland, and the breed has endangered status. Recognising the breed’s significance to Poland, the study aimed to monitor the occurrence of FFS in the Polish population.

## Material and Methods

Biological material from Silesian horses has been routinely collected since 2016 as part of a genetic disease monitoring programme for horses in Poland. A total of 284 horses born between 1994 and 2024 and being actively bred were evaluated (165 breeding mares and 116 stallions). The pedigree data were obtained from the official databases of the Polish Horse Breeders Association (PZHK). According to PZHK Silesian horse breeding statistics from 2021, there were 1,777 registered mares and 255 sires (8).

Extraction of DNA was undertaken from whole blood or hair follicles using the Sherlock AX kit (A&A Biotechnology, Gdansk, Poland), following the manufacturer’s instructions. Genotyping of the *PLOD1* c.2032G>A variant was carried out using a PCR and restriction-fragment length polymorphism analysis. Primers were designed based on the ENSECAG00000022842 reference sequence using Primer3 Input 0.4.0 (12). The forward primer sequence was 5′-CTCGTGGTAGTGCGTGAGTC-3′ and the reverse was 5′-AGGGCCCAGCTTCCTCTT-3′. The *FauI* restriction enzyme, which selectively cleaves the wild-type G allele but does not cut the mutant A allele, was identified using NebCutter V2.0 (13). Amplification by PCR was conducted with AmpliTaq Gold 360 Master Mix (Thermo Fisher Scientific, Waltham, MA, USA) under standard conditions and at an annealing temperature of 56°C. Digestion of PCR products with *Fau*I (New England Biolabs, Ipswich, MA, USA) was performed overnight at 60°C, and the resulting fragments were separated on a 4% agarose gel. Sanger sequencing was used to validate all heterozygous and a subset of homozygous samples, employing the BigDye Terminator v3.1 Cycle Sequencing Kit (Thermo Fisher Scientific) and a 3500xL Genetic Analyzer (Applied Biosystems, Foster City, CA, USA). Since no positive control was stored in the database of the National Research Institute of Animal Production, the selected samples were sent to University of California Davis (Davis, CA, USA) for method validation.

The basic statistics for allele frequencies (allele frequencies themselves, chi-squared test for Hardy–Weinberg equilibrium (HWE) and 95% confidence intervals (CI)) were calculated in SAS v.8.2 (SAS Institute, Cary, NC, USA), with P-values < 0.05 taken to be significant.

## Results

Digestion by *Fau*I yielded two cut fragments of 102 bp and 64 bp for the G allele and an uncut 166 bp fragment for the A allele, which facilitated genotype determination. In total, 29 horses (10.2%) among the 284 which were genotyped were identified as heterozygous for the FFS mutation (N/FFS), resulting in an allele frequency of 0.063 in the analysed population. The 95% confidence interval was 0.043–0.083. The chi-squared test for HWE yielded an χ^2^ value of 1.45, indicating that there was no significant deviation from Hardy–Weinberg equilibrium (Table 1.).

**Table 1.** Genotype analysis results for carriage of the mutation causing fragile foal syndrome (FFS) in Polish Silesian warmblood horses

n	Genotype totals							Genotype frequency		Allele frequency		95% CI range
	Observed	Expected*	Observed	Expected*	Observed	Expected*						
	N/N	N/N	N/FFS	N/FFS	FFS/FFS	FFS/FFS	N/N	N/FFS	FFS/FFS	N	FFS	
	248		36		0							
284							0.8732	0.1268	0	0.9366	0.0634	1.3005
		249.2		33.5		1.3						

* – calculated by chi-squared test for Hardy–Weinberg equilibrium; CI – confidence interval; n – number of analysed horses; N – wild-type allele; FFS – mutant allele

**Fig. 1.**
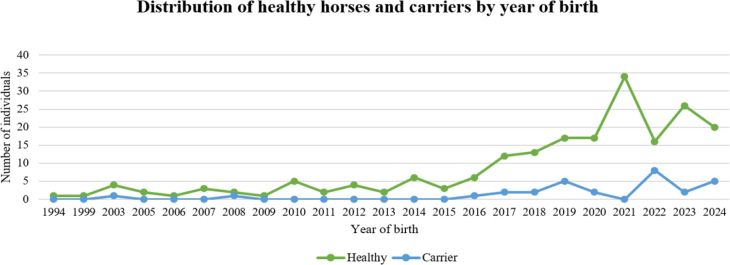
Birth-year breakdown of fragile foal syndrome gene carriage in the analysed Silesian horse population

The remaining 255 horses (89.8%) of the genotyped Silesian horses were identified as homozygous for the reference wild-type allele (N/N). No individuals homozygous for the lethal FFS mutation were found. However, no data were available regarding abortions or stillbirths. The number of horses genotyped across birth year cohorts varied substantially (Fig. 1).

**Fig. 2.**
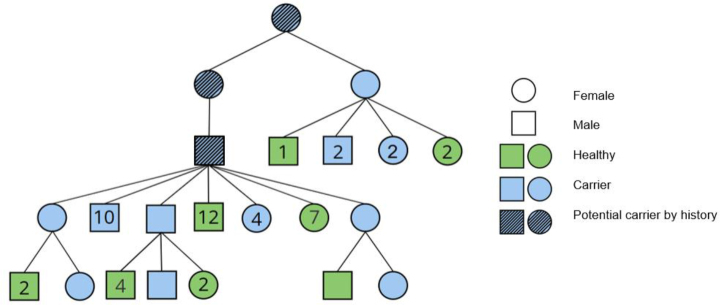
Pedigree chart showing inheritance of the mutation causing fragile foal syndrome in Polish Silesian horses. Numbers inside shapes are offspring totals

The first carrier was found to be a horse born in 2003, and the highest number of carriers were detected in the 2022 cohort. A pedigree analysis revealed that all horses carrying the mutation were related and shared a common ancestor (Fig. 2).

## Discussion

Genetic testing of domestic animals, including horses, is becoming increasingly popular and economically justified because it allows losses related to animal treatment and deaths to be avoided. The availability and low cost of testing, along with the ability to isolate DNA from biological material that does not require veterinary intervention to obtain, are making it an increasingly common component of breeding strategies.

In equines, the hereditary disease with symptoms of hyperelastic and abnormally fragile skin and joint hyperextensibility corresponds to Ehlers–Danlos syndrome (EDS) in humans (4). The newest classification of EDS in humans includes 13 types with over 20 mutations in various genes, mostly associated with collagen-related pathways (3, 7). In horses, two single nucleotide mutations have been identified as causative of EDS-like syndromes. The first occurs in the gene encoding cyclophilin B (*PPIB:* c.115 G>A). The disorder caused by this mutation is known as hereditary equine regional dermal asthenia and occurs in Quarter Horses and related breeds (11). The second is the mutation within the *PLOD* gene, and this is most common in warmblood horses and was investigated in the presented study. The obtained results showed allele frequency at 0.0634 and the number of carriers remaining consistently low over three decades, with only minor fluctuations. However, a slight increase can be observed in recent years, particularly in 2022. The study revealed that all carrier horses trace their lineage back to a single individual from the 1980s. The observed increase in carrier numbers results from the growing popularity of a prominent stallion (Figs 1 and 2). Previous large-scale screening across a wide range of breeds and many horses identified the presence of the mutated allele in numerous warmblood breeds, including the Silesian breed, which had a carrier allele frequency of 12.5% (9).The present study on a sample size of 284 horses showed that the carrier frequency was higher at 14.6%. Because of the conserved molecular background and clinical similarity to the human Ehlers–Danlos syndrome, FFS in horses can serve as a valuable spontaneous animal model for studying collagen-related connective tissue disorders.

When comparing the obtained results with those from other warmblood breeds from Europe, our estimates of carriage were higher than those among Swedish warmbloods (1), Trackeners, Westfalens and Selles Français (9). However, the frequency of carriers is similar to the frequency in Hanoverian warmbloods (5) and to that in Oldenburg horses, from which the Silesian horse’s origin is documented (9).

Breeders of Silesian horses need to be aware of this situation and take appropriate steps to minimise the risk of FFS in foals. Regular genetic testing and selective breeding can help reduce the number of carriers in future generations. Further research is necessary to develop effective breeding strategies aimed at reducing the frequency of carriers and improving the health of the Silesian horse population.

## Conclusion

This finding highlights the impact of selective breeding and the potential for unintentional propagation of genetic mutations through widely used sires. It underscores the importance of genetic monitoring in breeding programmes to prevent the spread of inherited disorders.
